# Control of cardiometabolic risk factors and their association with carotid intima media thickness among patients with type 2 diabetes mellitus-single center experience in a developing country

**DOI:** 10.55730/1300-0144.5821

**Published:** 2024-01-11

**Authors:** Thilak Priyantha WEERARATHNA, Sarath LEKAMWASAM, Iroshani KODIKARA, Keddegoda Gamage Piyumi WASANA, Lakmal FONSEKA

**Affiliations:** 1 Department of Medicine, Faculty of Medicine, University of Ruhuna, Galle, Sri Lanka; 2 Department of Anatomy, Faculty of Medicine, University of Ruhuna, Galle, Sri Lanka; 3 Diabetes Centre, Cooperative Hospital, Galle, Sri Lanka

**Keywords:** Atherosclerotic cardiovascular diseases, cardiometabolic risk factors, carotid intima-media thickness, type 2 diabetes mellitus

## Abstract

**Background/aim:**

Type 2 diabetes mellitus (T2DM) is closely associated with atherosclerotic cardiovascular diseases (ASCVD). The objective of this study was to describe the degree of ASCVD risk factor control and their association with carotid intima-media thickness (CIMT) in T2DM patients followed up at a diabetes clinic in Southern, Sri Lanka.

**Materials and methods:**

A crosssectional study was conducted to examine the association between CIMT and nonalcoholic fatty liver disease (NAFLD)in 300 T2DM patients. Both CIMT and its associations with modifiable cardiometabolic risk factors were examined using ultrasonography. The recommended optimal targets for risk factors were defined as glycated hemoglobin (HbA_1C_) < 7 %, absence of NAFLD, albumin-to-creatinine ratio (ACR) < 30 mg, triglyceride (TG) < 150 mg/dL, low-density lipoprotein cholesterol (LDL-C) < 100 mg/dL, high-density lipoprotein cholesterol (HDL-C) in men > 40 and in women > 50 mg/dL, systolic blood pressure (SBP) < 130 mmHg, and diastolic blood pressure (DBP) < 80 mmHg.

**Results:**

SBP, DBP, LDL-C, TG, HDL-C, HbA_1C_, and ACR were optimally controlled in 59.3%, 75.0%, 46.7%, 84.3%, 46.0%, 33.0%, and 18.7% of patients, respectively. Notably, nearly half of the study subjects did not have NAFLD. Only three patients (1%) had achieved all therapeutic targets. There were statistically significant differences in CIMT between optimally controlled TG and suboptimally controlled TG group (p = 0.027) and between the groups with and without NAFLD (p = 0.045) when adjusted for age and duration of diabetes. CIMT showed significant and positive associations with LDL-C (p = 0.024), TG (p = 0.026), and NAFLD (p = 0.005). Among these, the presence of NAFLD had the highest odds of having higher CIMT when compared to LDL-C and TG.

**Conclusion:**

The majority of patients have not achieved the recommended targets for ASCVD risk factors and are at high risk of ASCVD. It is therefore necessary to identify the reasons for not achieving the treatment targets in order to reduce the ASCVD burden by controlling LDL-C, TG, and NAFLD.

## 1. Introduction

Over the past few decades, there has been an exponential rise in morbidity and mortality caused by atherosclerotic cardiovascular diseases (ASCVD), and the pandemic of diabetes mellitus has substantially contributed to this upsurge [1,2]. Cardiovascular diseases (CVDs) account for nearly three-fourths of deaths in patients with type 2 diabetes mellitus (T2DM) [3]. To date, it has been shown that patients with T2DM are at a 2-fold higher risk for CVD compared to those without diabetes mellitus [4]. In the prospective Atherosclerosis Risk in Communities (ARIC) study, which followed up 13,790 patients over a two-year period, it was revealed that a patient with T2DM without established CVD has the same risk of developing a myocardial infarction as an individual who has already developed a myocardial infarction [5]. These observations have led to the notion that T2DM is a major determinant of CVD.

Several metabolic derangements seen in diabetes are responsible for both the initiation and progression of arterial injury, thereby increasing the risk of ASCVD observed among these patients. Hyperglycemia, insulin resistance coupled with hyperinsulinemia, atherogenic dyslipidemia, elevated systolic and diastolic blood pressures, and microalbuminuria operate at different molecular and cellular levels, increasing arteriopathy and the ASCVD risk in patients with T2DM [6]. Furthermore, chronic kidney disease resulting from diabetic nephropathy and comorbidities such as nonalcoholic fatty liver disease (NAFLD) are potential contributors to the increased ASCVD risk in patients with T2DM [6].

Epidemiological studies conducted in multiethnic settings have shown that patients with T2DM of South Asian ethnicity have a significantly higher risk of ASCVD compared to other ethnic groups [7]. Although several genetic, environmental, and behavioral factors have been postulated to explain the reported high risk of ASCVD among South Asian T2DM patients, the underlying mechanisms for this observation remain largely obscure.

Health authorities and professional organizations have embarked on several strategies to combat the rising incidents of death in patients with T2DM, especially due to ASCVD. They mainly focus on lifestyle and pharmacological manipulations of modifiable risk factors including dyslipidemia, microalbuminuria, hyperglycemia, and high blood pressure. Based on major prospective observational and interventional studies, optimal levels for these risk factors have been laid down as recommended therapeutic targets [8]. Major clinical trials and real-life studies provide evidence that adhering to these recommended therapeutic targets reduces adverse cardiovascular events in patients with T2DM [9]. The other primary prevention strategies include an early identification of arterial injury and implementation of appropriate therapeutic interventions to delay its progression. The impact of these strategies on the rising ASCVD burden, however, depends on several factors, including the awareness of caregivers to recommend appropriate interventions, the readiness of patients to follow them and achieve the recommended treatment targets, and the wider availability of resources to screen ASCVD at asymptomatic stages. Due to the variation of above factors, the degree of achievement of endorsed targets for ASCVD risk factors varies among T2DM patients living in different localities around the world [10].

Developing countries such as Sri Lanka are facing rising burden from T2DM and its related complications. These are reflected in the recent trends in community survey-based data, the country’s hospital admissions, and morbidity and mortality data [11,12]. Although these data have highlighted the rising burden of diabetes and related ASCVD on the healthcare settings and the community in Sri Lanka, there is a paucity of literature on the degree of cardiometabolic risk factor control and their associations with carotid intima-media thickness (CIMT), as a surrogate of chronic arterial injury, in patients with T2DM. Having knowledge about the intensity of control and strengths of associations among major modifiable ASCVD risk factors would enable clinicians to focus more on the most pivotal risk factors with strong associations, thereby reducing morbidity and mortality in T2DM patients, especially in the resource-poor settings in developing countries. Herein, we aimed to describe the degree of control of cardiometabolic risk factors and study their associations with CIMT in a cohort of patients with T2DM followed up at a tertiary care diabetes clinic in Southern, Sri Lanka.

## 2. Materials and methods

### 2.1. Study design and setting

The present study was designed as a single center crosssectional study. The study was conducted in the university medical clinic at the Teaching Hospital Karapitiya in Southern, Sri Lanka during January and June in 2021. Teaching Hospital Karapitiya, the largest tertiary care center in Southern, Sri Lanka, serves as the main training facility center for the Faculty of Medicine, University of Ruhuna, Sri Lanka. The university medical clinic receives referrals from other subspecialties in the hospital, outpatients’ departments, and primary and secondary care services in the area. All services, including drugs and investigations, are sponsored by the state and the hospital service area includes all ethnicities, almost in the same proportions as those found in the country.

### 2.2. Sample size and patients

The sample size was determined with the prevalence of CIMT among patients with T2DM as 57% and the margin of error of 6% [13].


$$\text{n}={\text{Z}}^{2}\text{P}\;(1-\text{P})/{\text{W}}^{2},$$

where, n = minimum sample size, Z = 1.96 (for 95% confidence interval). The calculated minimum sample size was 262; therefore, the final sample size was increased to 300 to account for missing data and incomplete questionnaires.

A total of 300 patients with T2DM, aged between 18 and 70 years, were enrolled for the study. The patients were attending the outpatient clinic of the University Medical Unit at the Teaching Hospital Karapitiya in Southern, Sri Lanka. Patients with established ASCVD, previous myocardial ischemia, stroke, transient ischemic attacks, peripheral arterial disease, vascular dementia, and type 1 diabetes mellitus were excluded from the study. Pregnant women were also excluded. An identification number was assigned to each study subject at the time of data collection. All the data collected during the study were stored on the principal investigator’s laptop and in a data repository with respect to their identification numbers. Data were not shared with individuals other than the investigators of the study, and all investigators had access to the data repository.

### 2.3. Variables

According to the objectives of the present study, the primary outcome was to determine the degree of control of cardiometabolic risk factors, including glycated hemoglobin (HbA_1C_), absence of NAFLD, albumin-to-creatinine ratio (ACR), triglyceride (TG), low-density lipoprotein cholesterol (LDL-C), high-density lipoprotein cholesterol (HDL-C), systolic blood pressure (SBP), and diastolic blood pressure (DBP) in T2DM patients. The secondary outcome was to estimate the associated cardiometabolic risk factors with CIMT in a cohort of patients with T2DM. Age, sex, and the duration of T2DM were the potential confounders for the outcomes.

### 2.4. Laboratory and demographic measurements

A fasting venous blood sample (5 cc) was collected and HbA_1C_, serum creatinine, serum albumin, and lipid profile including LDL-C, TG, and HDL-C were measured. HbA_1C_ was measured by HPLC method using an automated analyzer (Bio-Rad D analyzer, Bio-Rad, Hercules, CA, USA) while lipid profile, serum creatinine, and albumin were measured by a fully automated analyzer based on spectrophotometric principles (HumaStar 600 HS, Human Diagnostics, Wiesbaden, Germany). Estimated globular filtration rate (eGFR) was calculated in mL/min/1.73 m^2^ using the Chronic Kidney Disease Epidemiology Collaboration equation. ACR was used to measure the amount of albumin in the urine. All the biochemical estimations were performed at the same laboratory with periodic in vitro quality control measures and results were entered into a data entry form.

Demographic data including sex, age, duration of diabetes were collected. Body weight, height, and waist circumference (WC) were measured, and thus, body mass index (BMI) was calculated as the body weight (kg) divided by the height squared (m^2^). Both SBP and DBP were recorded as an average of three readings.

The ultrasonographic assessment of the liver was performed by a specialist radiologist (IK) using the curvilinear probe (3.5 MHz) of the high-end ultrasound unit (GE LOGIQ E9 XDclear, GE HealthCare, Seongnam, Gyeonggi, Korea) to detect NAFLD. Upon the diagnosis of fatty liver, an increase in liver echogenicity was observed compared to the renal cortex and spleen. Additionally, there was a loss of definition of the diaphragm and poor delineation of the intrahepatic portal venous architecture. Achievement of one or two of the mentioned criteria was required to prevent false positive results. The status of NAFLD was documented as present or absent.

Ultrasonographic measurement of CIMT was performed using the linear transducer (3–11 MHz) of GE LOGIQ E9 XDclear scanner. The best quality images were obtained by adjusting the time gain compensation curve and amplifying the grayscale. A single focus point was adjusted at the level of the posterior wall of the carotid artery and in optimum magnification, the measurements were taken from the posterior wall. Both common carotid arteries in a plaque-free segment were used to measure CIMT. The average of three measurements was then taken as the CIMT. The mean value of both CIMT measurements was subsequently employed for the analyses.

### 2.5. Statistical analysis

After having checked the data sets for distribution, the normally distributed data sets were expressed as mean ± standard deviation (SD). Skewed data sets were represented in median value with the interquartile range. All categorical data were reported as numbers with the percentage. Optimal levels of cardiometabolic risk factors were defined according to the American Diabetes Association (ADA) clinical practice guidelines (HbA_1C_ < 7 %, absence of NAFLD, ACR < 30 mg, TG < 150 mg/dL, LDL-C < 100 mg/dL, HDL-C in males > 40 and in females > 50 mg/dL, SBP < 130 mmHg, and DBP < 80 mmHg). The one-way ANCOVA was applied to determine whether there were significant differences of CIMT between each optimally and suboptimally controlled groups, separately. In the one-way ANCOVA, age and the duration of T2DM were used as the covariates. The strength of correlations between CIMT and the risk factors were determined using either Pearson correlation coefficient or Spearman’s rank correlation coefficient. A point-biserial correlation was applied to measure the correlation between CIMT and NAFLD. Association between CIMT and the risk factors was assessed through multiple linear regression at 95% CI with the adjustment for age, sex, and the duration of T2DM. Missing values were not inferred for the study analysis. A significance level of p ≤ 0.05 was considered. All the analyses were carried out using SPSS version 25.0 (IBM Corporation, Armonk, NY, USA).

## 3. Results

**Figure 1:**
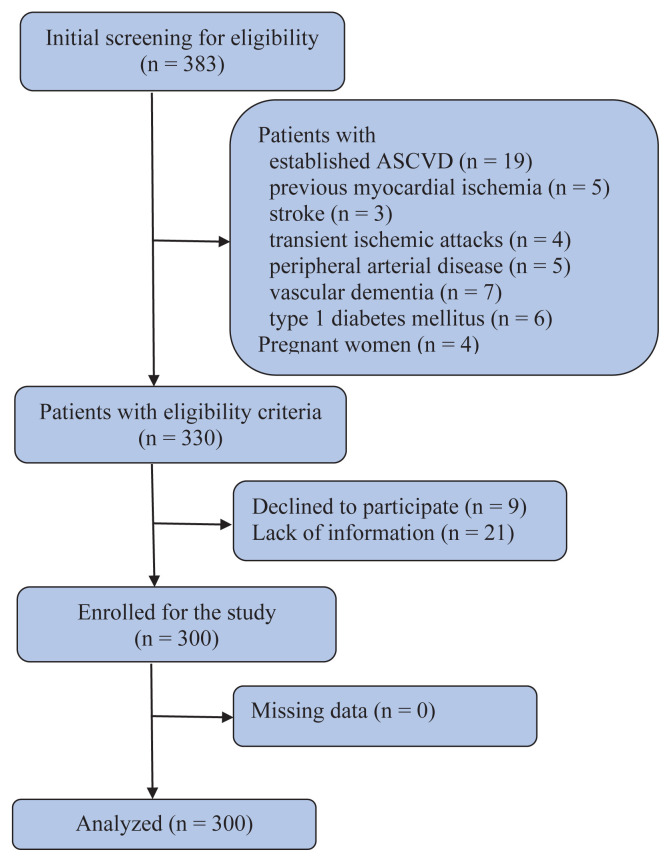
Study participants’ flow diagram, from recruitment to analysis.

A total of 330 T2DM patients were eligible for the study and among them, 300 patients (209 women) were enrolled for the study (Figure 1). Table 1 includes the basic characteristics of the study subjects.

Of all 300 patients, only three patients (1%) had all cardiometabolic risk factors controlled at optimal levels. The therapeutic targets of risk factors such as SBP, DBP, LDL-C, TG, HDL-C, HbA_1C_, and ACR were achieved by 59.3%, 75.0%, 46.7%, 84.3%, 46.0%, 33.0%, and 18.7% of patients, respectively. Nearly half of the study subjects were found to have NAFLD (Table 2). Furthermore, mean CIMTs were significantly different between those with optimally controlled TG and those without, as well as between those with and without NAFLD when adjusted for age and the duration of T2DM (Table 2).

**Figure 2:**
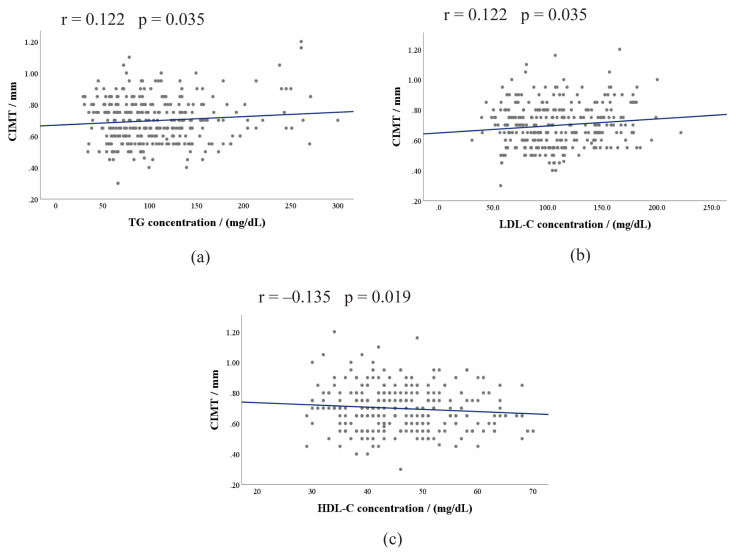
Correlation matrix plots of CIMT with (a) TG, (b) LDL-C, and (c) HDL-C. CIMT, carotid intima-media thickness; HDL-C, high-density lipoprotein cholesterol; LDL-C, low-density lipoprotein cholesterol; TG, triglyceride.

In study subjects, TG and LDL-C showed significant and positive correlations with CIMT (Figures 2a and 2b) while HDL-C showed an inverse correlation (Figure 2c). SBP, DBP, HbA_1C_, ACR, as well as the duration of T2DM, however, showed no significant correlations with CIMT in these patients. Furthermore, a positive correlation was observed between NAFLD and CIMT (r = 0.161, p = 0.005).

As revealed by multiple linear regression analysis (Table 3), CIMT showed significant and positive associations with LDL-C (p = 0.024), TG (p = 0.026), and NAFLD (p = 0.005). The presence of NAFLD had the highest odds of having higher CIMT when compared to LDL-C and TG.

## 4. Discussion

This single center investigation exposed several important facts regarding the control of major amendable cardiometabolic risk factors and their association with CIMT in a cohort of adults with T2DM without established cardiovascular disease, in a developing South Asian country.

First, these findings exposed the alarming suboptimal control of major amendable ASCVD risk factors among patients with T2DM in a tertiary care center in Sri Lanka, a country facing a rising burden of CVD. Only about one third (33%) of patients have achieved the recommended therapeutic targets of glucose control determined by HbA_1C_, while only 46% have achieved the optimal LDL-C target. In contrast, a high proportion of patients had achieved systolic and diastolic blood pressures targets (75% and 59%, respectively). Furthermore, optimal control of HbA_1C_, all major lipid, and blood pressure parameters was achieved by only a small fraction (1%) of study participants. Secondly, these findings reconfirm the established associations of high LDL-C, TG, and low HDL-C with CIMT. However, the stronger association of NAFLD with CIMT, compared to LDL-C, TG, and HDL-C, is a somewhat novel finding emerging from this analysis.

In the real-world setting, only a small proportion of diabetic patients achieve the recommended targets for the control of major CVD risk factors. According to a multicenter survey conducted in China involving T2DM patients, optimal glycemic control was observed in only 32.6% of patients, while the control of lipids, glycemia, and blood pressure together was achieved in only 11.2% [14]. Wong et al. (2013) reported that only 24% of US adults with T2DM control all three CVD risk factors, including LDL-C, HbA_1C_, and blood pressure [15]. In a recent metaanalysis, the achievement rates for the control of CVD risk factors—glycemic control, blood pressure, LDL-C, HDL-C, and TG—were 42.8%, 29%, 49.2%, 58.2%, and 61.9% respectively [16]. During 2013 and 2017, control rates of HbA_1C_, LDL-C, and blood pressure had increased among hospitalized T2DM patients in Tianjin, China [17]. A crosssectional study conducted in a tertiary care teaching hospital in Wardha District, India, demonstrated that only one-fourths of T2DM patients have the optimal control of one or more risk factors for CVD, including physical inactivity, tobacco use, hypertension, obesity, dyslipidemia, and dietary risk factors [18]. A previous crosssectional study carried out at a private diabetes center in Sri Lanka revealed that the percentage of patients achieving the recommended therapeutic targets for CVD risk factors was 25.2% for HbA_1C_, 24.3% for LDL-C, 32% for SBP, and 56.7% for DBP [19].

Atherosclerosis is a chronic process initiated by arterial injury due to a variety of factors, progressing over decades. There is an imperative need for a low-cost, convenient, and noninvasive screening tool to detect progressive arteriopathy before its adverse clinical outcomes occur, especially among at-risk populations such as those with T2DM. CIMT is a reliable and noninvasive indicator of chronic arterial injury [20]. Similar to the current study findings, Li et al. [21] found a significant association between TG with CIMT among 1476 T2DM patients in Affiliated Hospital of Southwest Medical University. However, they found an association between blood glucose and CIMT in diabetic patients, whereas our analysis did not find such an association between HbA_1C_ and CIMT. In accordance with our findings, Kowall et al. [22] also observed that the measures of glycaemia were not associated with CIMT in diabetic and nondiabetic subjects in Southern Germany (n = 2663). However, HbA_1C_ was found to be a significant determinant of CIMT in nondiabetic community dwelling individuals [23]. Although some studies have found significant associations of CIMT with SBP, DBP, and ACR [21,23–25], our study failed to observe such associations. This could partly be due to obtaining office measurement of SBP, DBP, and ACR in our study. Office blood pressure measurement often does not reflect the control of blood pressure over a period, and patients may have been more compliant with medication before the scheduled clinic visit.

South Asians possess a lipid profile that favors chronic arterial injury, especially with high TG and low HDL-C, compared to other ethnicities [26–28]. We observed a significantly higher CIMT in T2DM patients with suboptimally controlled TG. However, there were no significant associations between CIMT and other lipids, namely LDL-C and HDL-C, and this could be due to several reasons. South Asians are reputed to have arterial injuries at relatively lower levels of LDL-C [26]. However, despite having high HDL-C levels (>40 mg/dL), a considerable percentage of South Asians experience dysfunctional in their HDL-C. Indeed, dysfunctional HDL-C has fewer defensive aptitudes and proinflammatory effects, thereby linking with the progression of CVD [29].

There is increasing prevalence of NAFLD globally, and it is estimated to affect more than half of T2DM patients [30]. The leading cause of mortality in patients with NAFLD is CVD. A recently study involving 3000 patients demonstrated a significantly higher prevalence of CVD in T2DM patients with ultrasound diagnosed NAFLD compared to those without NAFLD [31]. It was reported that there is a 90% increase in the risk of CVD risk in T2DM patients with NAFLD diagnose via ultrasonography [32]. A large metaanalysis reconfirmed that NAFLD is a risk factor for both fatal and nonfatal cardiovascular events among patients with T2DM [33]. An investigation undertaken in China involving study participants (n = 71,852) who had no previous cardiovascular events demonstrated that NAFLD is an independent predictor of CVD not only in patients with T2DM but also in those with prediabetes [34]. A prospective pilot study, including 529 T2DM outpatients with no history of CVD, has further reported that NAFLD detected via nonenhanced computed tomography could be used to identify T2DM patients at high risk for CVD [35]. In addition to the aforementioned studies, several investigations have highlighted the association between NAFLD and CVD in patients with T2DM [36,37].

Notably, it is reported that CVD accompanied by NAFLD exerts a more adverse metabolic burden than CVD alone [38]. In fact, the strong association between NAFLD and CVD is governed by numerous pathophysiological mechanisms, including oxidative stress, inflammation, lipid metabolism, and gut microbiota, etc. [39–41]. In NAFLD patients, higher levels of TG, LDL-C, and VLDL-C, which are established ASCVD risk factors, can be observed due to increased lipogenesis and the overflow of free fatty acids to the liver [42–44]. Furthermore, increased overflow of free fatty acids to the liver leads to oxidative stress, which induces endothelial dysfunction. Ultimately, this whole process enhances CVD events [45,46]. Additionally, hepatic insulin resistance and inflammation accelerate CVD risk [47,48]. Moreover, the increase in intestinal microbiota could influence lipid metabolism, adipose inflammation, and insulin resistance, which are collective mechanisms linking NAFLD and CVD events [49]. In the present study, NAFLD was found to be a more significant marker of chronic arterial injury, as measured by CIMT, compared to other conventional risk factors such as LDL-C and TG.

The main strength of the present study is assessing CIMT in patients before the onset of adverse ASCVD clinical outcomes such as myocardial and cerebrovascular disease. Furthermore, we considered all major modifiable ASCVD risk factors to draw final conclusions. Nevertheless, it is necessary to address certain limitations. This study was a single center, crosssectional study involving only 300 T2DM patients, which limits the generalizability of our findings. We propose further studies with a wider inclusion of study centers and more study participants. It should also be noted that we assessed the severity of chronic arterial injury in these patients by measuring their CIMT, which is considered a less robust indicator of atherosclerosis compared to other measurements such as quantification of plaque burden or coronary artery calcium score. However, in the low-resource setting, we were compelled to use CIMT as a surrogate for arterial injury. The potential confounding effects of antidiabetic, antihyperlipidemic, and antihypertensive treatments on our findings were also not investigated in this study. Furthermore, NAFLD was detected in dichotomous manner—present or absent—disregarding the different stages of fat content. Additionally, ultrasonography, compared to MRI, has low sensitivity and specificity in differentiating the different stages of fatty liver.

In conclusion, the control of major modifiable ASCVD risk factors—SBP, DBP, LDL-C, TG, HDL-C, HbA_1C_, and ACR—by patients with T2DM is grossly suboptimal in the present cohort. Significantly higher CIMT was observed in T2DM patients with suboptimal control of TG compared to those with optimal control of TG. Importantly, T2DM patients with NAFLD had higher CIMT than those without NAFLD.

The lipid profile parameters, including HDL-C, LDL-C, and TG, as well as NAFLD, were found to be significantly correlated with CIMT in the study subjects. This study indicates that LDL-C, TG, and the presence of NAFLD are significant predictors of chronic arterial injury in patients with T2DM. In general, these findings could be utilized to strengthen the current care delivered by the study center by educating patients as well as doctors involved in patient care. Furthermore, this data could be used as a platform in designing future studies.

## Figures and Tables

**Table 1:** Patient and disease related variables of 300 patients included in the analysis.

Variable	Results
Number (%) of women	209 (69.7)
Mean ± SD age (years)	62 ± 10
Median (IQR) of the duration of T2DM (years)	7 (4–11)
Mean ± SD BMI (kg/m^2^)	24.4 ± 3.8
Median (IQR) WC (cm)	87.0 (82.0–95.0)
Median (IQR) systolic pressure mmHg	126.0 (114.0–140.0)
Median (IQR) diastolic pressure mmHg	72.0 (63.0–80.8)
Median (IQR) TG (mg/dL)	96.0 (71.0–133.8)
Median (IQR) HDL-C (mg/dL)	45.0 (39.3–51.0)
Median (IQR) LDL-C (mg/dL)	104.6 (79.0–140.0)
Median (IQR) HbA_1C_ (%)	7.65 (6.80–9.00)
Mean ± SD eGFR (mL/min/1.73 m^2^)	73.4 ± 23.6
Median (IQR) ACR (mg/mmol)	68.8 (35.1–120.1)
Mean ± SD CIMT (mm)	0.70 ± 0.14
Number (%) with NAFLD	147 (49.0)

ACR, urine albumin-to-creatinine ratio; BMI, body mass index; CIMT, carotid intima-media thickness; eGFR, estimated glomerular filtration rate; HbA_1C,_ glycated hemoglobin; HDL-C, high-density lipoprotein cholesterol; LDL-C, low-density lipoprotein cholesterol; NAFLD, nonalcoholic fatty liver disease; TG, triglyceride; T2DM, type 2 diabetes mellitus; WC, waist circumference.

**Table 2:** Mean differences of CIMT based on the control of cardiometabolic risk factors.

Factor	State	No. of study subjects (%)	CIMT/mm Mean ± SD	p value
SBP	Optimal	178 (59.3)	0.69 ± 0.14	0.87
	Suboptimal	122 (40.7)	0.71 ± 0.14	
DBP	Optimal	225 (75.0)	0.70 ± 0.14	0.70
	Suboptimal	75 (25.0)	0.69 ± 0.13	
LDL-C	Optimal	140 (46.7)	0.69 ± 0.13	0.11
	Suboptimal	160 (53.3)	0.70 ± 0.15	
TG	Optimal	253 (84.3)	0.69 ± 0.13	0.027
	Suboptimal	47 (15.7)	0.74 ± 0.17	
HDL-C	Optimal	140 (46.7)	0.68 ± 0.13	0.18
	Suboptimal	160 (53.3)	0.71 ± 0.15	
HbA_1C_	Optimal	99 (33.0)	0.69 ± 0.15	0.28
	Suboptimal	201 (67.0)	0.70 ± 0.14	
ACR	Optimal	56 (18.7)	0.67 ± 0.13	0.16
	Suboptimal	244 (81.3)	0.70 ± 0.14	
NAFLD	Absent	153 (51.0)	0.68 ± 0.14	0.045
	Present	147 (49.0)	0.72 ± 0.14	

ACR, urine albumin-to-creatinine ratio; CIMT, carotid intima-media thickness; HbA_1C_, glycated hemoglobin; HDL-C, high-density lipoprotein cholesterol; LDL-C, low-density lipoprotein cholesterol; NAFLD, nonalcoholic fatty liver disease; TG, triglyceride; WC, waist circumference.

**Table 3:** Associated risk factors with CIMT in patients with T2DM.

	OR (95% CI)	p value
HDL-C	−0.074 (−0.003 to 0.001)	0.20
LDL-C	0.131 (0.000–0.001)	0.024
TG	0.131 (0.000–0.001)	0.026
NAFLD	0.156 (0.014–0.074)	0.005

HDL-C, high-density lipoprotein cholesterol; LDL-C, low-density lipoprotein cholesterol; NAFLD, nonalcoholic fatty liver disease; TG, triglyceride.
